# Interpretable generalized neural additive models for mortality prediction of COVID-19 hospitalized patients in Hamadan, Iran

**DOI:** 10.1186/s12874-022-01827-y

**Published:** 2022-12-31

**Authors:** Samad Moslehi, Hossein Mahjub, Maryam Farhadian, Ali Reza Soltanian, Mojgan Mamani

**Affiliations:** 1 Department of Biostatistics, School of Public Health, Hamadan University of Medical Sciences, Hamadan, Iran; 2 Department of Biostatistics, School of Public Health, Research Center for Health Sciences, Hamadan University of Medical Sciences, Hamadan, Iran; 3 Department of Biostatistics, School of Public Health, Modeling of Noncommunicable Diseases Research Center, Hamadan University of Medical Sciences, Hamadan, Iran; 4 Brucellosis Research Center, Hamadan University of Medical Sciences, Hamadan, Iran

**Keywords:** COVID-19, Feature selection, Laboratory markers, Machine learning, Generalized neural additive, Prediction

## Abstract

**Background:**

The high number of COVID-19 deaths is a serious threat to the world. Demographic and clinical biomarkers are significantly associated with the mortality risk of this disease. This study aimed to implement Generalized Neural Additive Model (GNAM) as an interpretable machine learning method to predict the COVID-19 mortality of patients.

**Methods:**

This cohort study included 2181 COVID-19 patients admitted from February 2020 to July 2021 in Sina and Besat hospitals in Hamadan, west of Iran. A total of 22 baseline features including patients' demographic information and clinical biomarkers were collected. Four strategies including removing missing values, mean, K-Nearest Neighbor (KNN), and Multivariate Imputation by Chained Equations (MICE) imputation methods were used to deal with missing data. Firstly, the important features for predicting binary outcome (1: death, 0: recovery) were selected using the Random Forest (RF) method. Also, synthetic minority over-sampling technique (SMOTE) method was used for handling imbalanced data. Next, considering the selected features, the predictive performance of GNAM for predicting mortality outcome was compared with logistic regression, RF, generalized additive model (GAMs), gradient boosting decision tree (GBDT), and deep neural networks (DNNs) classification models. Each model trained on fifty different subsets of a train-test dataset to ensure a model performance. The average accuracy, F1-score and area under the curve (AUC) evaluation indices were used for comparison of the predictive performance of the models.

**Results:**

Out of the 2181 COVID-19 patients, 624 died during hospitalization and 1557 recovered. The missing rate was 3 percent for each patient. The mean age of dead patients (71.17 ± 14.44 years) was statistically significant higher than recovered patients (58.25 ± 16.52 years). Based on RF, 10 features with the highest relative importance were selected as the best influential features; including blood urea nitrogen (BUN), lymphocytes (Lym), age, blood sugar (BS), serum glutamic-oxaloacetic transaminase (SGOT), monocytes (Mono), blood creatinine (CR), neutrophils (NUT), alkaline phosphatase (ALP) and hematocrit (HCT). The results of predictive performance comparisons showed GNAM with the mean accuracy, F1-score, and mean AUC in the test dataset of 0.847, 0.691, and 0.774, respectively, had the best performance. The smooth function graphs learned from the GNAM were descending for the Lym and ascending for the other important features.

**Conclusions:**

Interpretable GNAM can perform well in predicting the mortality of COVID-19 patients. Therefore, the use of such a reliable model can help physicians to prioritize some important demographic and clinical biomarkers by identifying the effective features and the type of predictive trend in disease progression.

**Supplementary Information:**

The online version contains supplementary material available at 10.1186/s12874-022-01827-y.

## Background

Since late 2019, the spread of SARS-CoV-2 pneumonia, known as COVID-19, began in Wuhan, China, and has become a worldwide pandemic disease [1]. In the treatment of COVID-19 patients, the assessment of demographic, laboratory biomarkers, and clinical risk factors as well as the identification of death predictors in these patients have always been considered as one of the challenges facing researchers [2]. Several studies have been performed to evaluate changes in levels and relationships between laboratory biomarkers such as aspartate aminotransferase (AST), alanine aminotransferase (ALT), lymphocytes (LYM), neutrophil (NEU), and lactate dehydrogenase (LDH) in patients with COVID-19 [3, 4].

Recently, advanced models of medical information analysis have been extended that can help interpret complex biological relationships between clinical measurements and patient outcomes. Machine learning is a very powerful tool for identifying patterns, classifying clinical decision making, also identifying features of medical data that are relevant to clinical outcomes [5].

Prediction and classification models are designed to help healthcare professionals with some decisions such as using different diagnostic, starting or stopping treatments, using the available resources in a good way, and also can avoid some common biases in clinical decision making [6]. To estimate the probability that a specific outcome i.e. death will occur, risk prediction models are employed. These models used patient characteristics and the accuracy of the prediction depends on the ability of the model in discovering the complex relationship between patient characteristics and outcome [7, 8]. Recently, various machine learning methods have been used to predict and classify caused by COVID-19 mortality, including the use of logistic regression, RF, and GBDT [9–11].

To improve the performance of any analysis such as identifying the most important features and classification analysis many preprocessing techniques can be applied. One of the most important stages of preprocessing is dealing with missing values in features. Some methods require complete data without missing values. Conducting the analysis without considering missing values will bias the results and make some analyzes impossible [12]. There are different strategies in dealing with missing values such as deleting missing cases, but may leads to bias, imputing missing values using statistical imputation methods i.e., univariate methods; the mode, mean, or zero, but the results are not optimal, imputing missing values with KNN, and MICE imputation methods [13–16].

In most cases, the relationship between features and the clinical outcomes is non-linear. For this situation, the classic models e.g. the linear regression models are inappropriate. There are methods such as GAMs that enable to capture of non-linear patterns [17]. GAMs are superior in several respects, and the purpose of using these models is to maximize the accuracy of response prediction and to discover the nonlinear relationships of predictor features while maintaining explain ability [18].

Machine learning algorithms seem to be suitable as a nonlinear method for data modeling as well as for predicting and classifying responses, because of automatic discovering the relationships between the data and being able to generate a suitable output with minimum error [19]. Among the machine learning methods that have been used for prediction, we can mention neural network-based methods such as DNNs, and GNAMs [20, 21].

Despite the remarkable results of DNNs in predicting the effects of clinical biomarkers on virus infections [22] and biomedical studies [23], since these models are considered as black-box models, it is inexplicit to understand how they perform their predictions and how can be interpreted. Therefore lack of interpretability is an inevitable problem in applying these methods in fields such as healthcare. Interpretive machine learning method inspired by generalized additive models is an emerging research topic that seeks to solve this problem.

One of the suitable methods for debugging neural network predictions is the use of GNAMs which are inherently interpretable. Advantages of GNAMs include showing a larger class of classic GAMs, interpretable of a neural network model, and showing learned diagrams as accurate descriptions of predictions [20].

This study aimed to predict COVID-19 patient outcomes (dead/recovered) admitted to hospitals in Hamadan, Iran, GAMs will be used as a classical method and DNNs and GNAMs will be used as machine learning methods. The fitted models will be compared with Accuracy and AUC classification indices. The behavior of each feature in mortality risk will be visualized and interpreted based on GNAMs.

## Methods

The method section is assigned into several subsections. First, data details used in this study was introduced in the subsection COVID-19 dataset. Then, in the subsection Data Imputation, imputation algorithms were introduced. In the subsection Data Description, the method of reporting the results is presented. In the subsection Feature Selection using random forest, the method of feature selection using the random forest algorithm was described. Finally, the classification models used in this study were fully explained in the Logistic regression model, Gradient Boosting Decision Tree (GBDT), Generalized Additive Models, Deep Neural Networks (DNN), and Generalized Neural Additive Models sub-sections. At last, in the Evaluation metrics and Class imbalanced issue subsection performance of the models was described.

### COVID-19 dataset

In this cohort study, the dataset of 2181 Covid-19 patients who were admitted to Sina (COVID-19 treatment center) and Besat hospitals affiliated to Hamadan University of Medical Sciences, Iran were used. In this study, patients with positive real time reverse transcriptase polymerase chain reaction (RT-PCR) on samples from upper respiratory nasopharyngeal swabs were enrolled to the study. The study was approved by the Ethical Committee of the Hamadan University of Medical Science with the approved ethical code: IR.UMSHA.REC.1400.366. The dataset was collected from patient information from February 2020 to July 2021, which includes baseline demographic and clinical biomarkers. Demographic characteristics i.e. age, sex, smoking, compromised immune system (Com.immune.sys), renal insufficiency, diabetes, and hypertension as well as clinical biomarkers i.e. erythrocyte sedimentation rate (ESR), blood urea nitrogen (BUN), blood sugar (BS), blood creatinine (CR), prothrombin (PT), serum glutamic-pyruvic transaminase (SGPT), serum glutamic-oxaloacetic transaminase (SGOT), alkaline phosphatase (Alp), thromboplastin or partial thromboplastin time (PTT), platelets (Plat), hematocrit (HCT), hemoglobin (Hb), lymphocytes (Lym), monocytes (Mono), and neutrophils (NUT) were collected from patient information. A total of 22 features (or input features) were retrieved, consisting of 15 clinical biomarkers and 7 demographic characteristics of patients. For all classification models the patient's recovery status considered as a binary outcome (death = 1 and recovery = 0).

### Data Imputation

The missing rate in this study was 3 percent based on all features for each patient. A detailed description of the missing rate for each feature was reported in Table 1. In this study, we followed four different strategies to deal with missing data. First, discarding entire rows (cases) containing missing values and subsequent analysis was done. In this strategy, the information of 2117 patients was analyzed (Complete case dataset). In the three other strategies missing values were imputed by the mean, the KNN imputation, the MICE method, and subsequent analysis was done (Imputed dataset). In the mean imputation method, the missing values in features are imputed by the average of all the observations in that feature that are not missing. In the KNN imputation method, the missing values are imputed by an average of the corresponding values of the k nearest features which are computed by similarity measures such as Euclidean distance. In this study, k varies from 5 to 100 and the best k value for imputation was 10. The MICE imputation method based on fully conditional specification, first, calculates the mean of each feature that has a missing value and uses the mean as replacement values. Then (linear/logistic) regression models with chain equations are fitted using features with missing values and target feature. Finally, the missing values are predicted and updated with 100 iterations.

**Table 1:** Descriptive statistic of demographic characteristics and laboratory biomarker of COVID-19 patients based on the complete dataset

**Categorical Feature**	Treatment Result		Total (N = 2181)	Missing rate (%)	P-value
	Recovered (N = 1557)	Dead (N = 624)			
	Frequency (%)	Frequency (%)	Frequency (%)		
Sex				0	
Male	819(52.6)	349(55.9)	1168(53.6)		0.125
Female	738(47.4)	275(44.1)	1013(46.4)		
Smoking				0	
Yes	114(7.3)	51(8.2)	165(7.6)		0.422
No	1443(92.7)	573(91.8)	2016(92.4)		
Com.immune.sys				0	
Yes	4(0.3)	1(0.2)	5(0.2)		0.675
No	1553(99.7)	623(99.8)	2176(99.8)		
Renal insufficiency				0	
Yes	65(4.2)	31(5.0)	96(4.4)		0.391
No	1492(95.8)	593(95.0)	2085(95.6)		
Diabetes				0	
Yes	314(20.2)	155(24.8)	469(21.5)		0.010
No	1243(79.8)	469(75.2)	1712(78.5)		
Hypertension				0	
Yes	509(32.7)	285(45.7)	749(36.4)		< 0.001
No	1048(67.3)	339(54.3)	1387(63.6)		
**Continues Feature**	Mean (± SD)	Mean (± SD)	Mean (± SD)		*P*-value
Age(year)	58.25(16.52)	71.17(14.44)	61.95(16.99)	0.05	< 0.001
ESR(mm/hr)	42.27(26.54)	47.53(31.31)	43.77(28.08)	0.23	< 0.001
BUN(mg/dl)	18.45(13.04)	32.97(25.15)	22.6(18.58)	0.23	< 0.001
BS(mg/dl)	139.05(72.25)	146.53(91.03)	141.17(78.08)	1.47	0.047
CR(mg/dl)	1.13(0.99)	1.59(1.25)	1.27(1.1)	0.23	< 0.001
PT(sec)	12.49(4.20)	13.72(4.83)	12.84(4.42)	1.33	< 0.001
SGPT(U/L)	34.70 (35.88)	49.50(59.46)	38.87(44.32)	1.42	< 0.001
SGOT(U/L)	38.89 (34.31)	65.54(67.96)	46.4(47.84)	1.38	< 0.001
ALP(U/L)	177.62(92.42)	231.0481(184.6)	192.82(127.76)	1.51	< 0.001
PTT(sec)	33.64(11.78)	34.91(14.34)	34(12.56)	1.28	0.067
Plat(× 1000µL)	196.83(80.27)	189.78(88.13)	194.82(82.64)	0.28	0.054
HCT(%)	42.28(5.80)	41.49(7.83)	42.06(6.46)	0.18	0.005
Hb(mg/l)	13.86(2.39)	13.42(2.70)	13.74(2.49)	0	< 0.001
LYM(%)	22.57 (11.49)	13.47(10.29)	19.95(11.9)	1.28	< 0.001
Mono(%)	3.06(2.39)	2.39(1.42)	2.87(2.13)	1.6	< 0.001
NUT(%)	72.58(12.01)	76.97(16.60)	73.85(13.65)	1.28	< 0.001

*Com.immune.sys* Compromised immune system, *Renal insuf* Renal insufficiency, *ESR* Erythrocyte Sedimentation Rate, *BUN* Blood Urea Nitrogen, *BS* Blood Sugar, CR Blood Creatinine, *PT* Prothrombin time, *SGPT* Serum Glutamic-Pyruvic Transaminase, *SGOT* Serum Glutamic-Oxaloacetic Transaminase, *Alp* Alkaline Phosphatase, *PTT* Partial Thromboplastin Time, *Plat* Platelets, *HCT* Hematocrit, *Hb* Hemoglobin, *Lym* Lymphocytes, *Mono* Monocytes, *NUT* Neutrophils

**Fig. 1:**
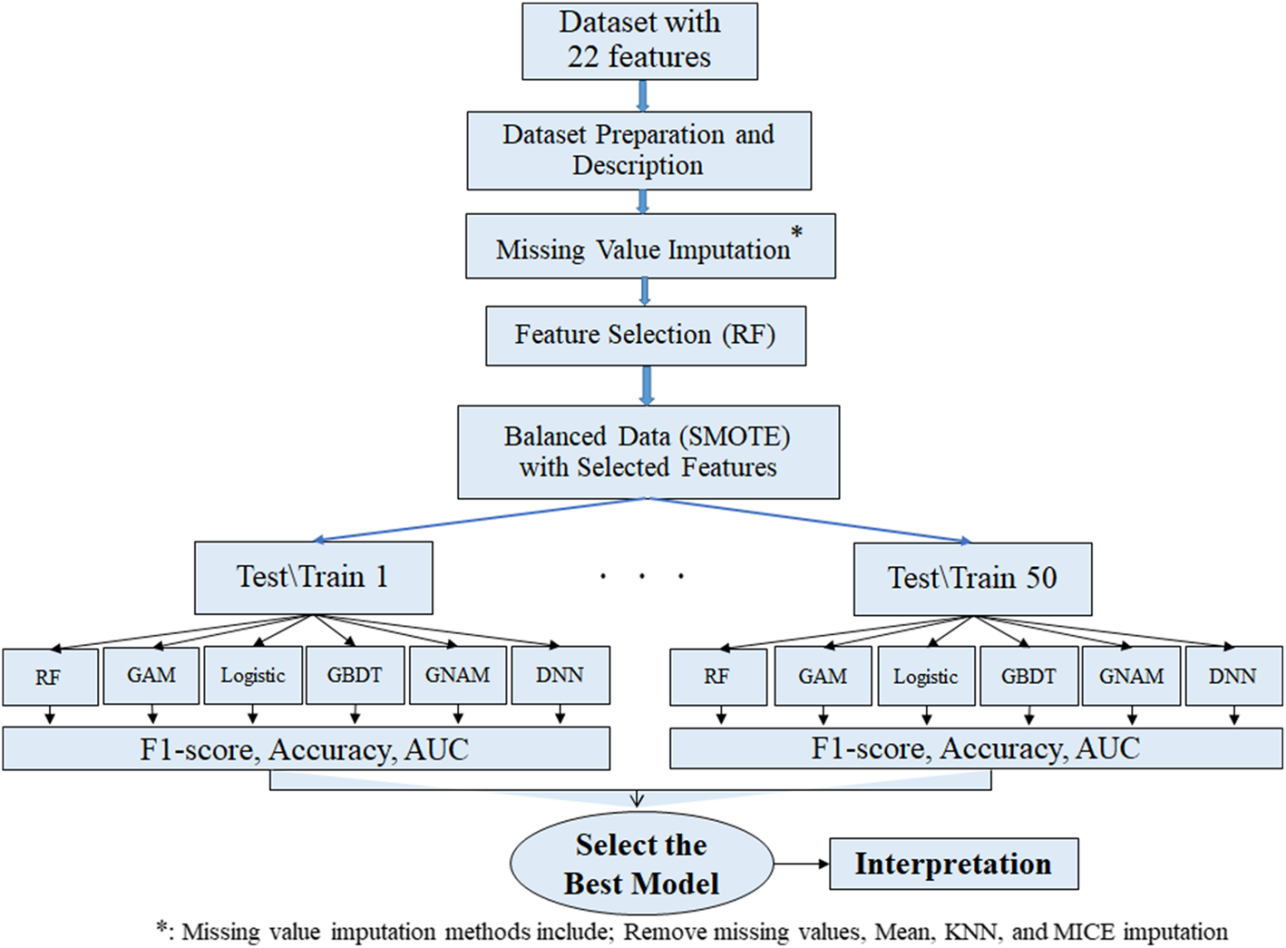
The steps of study design for COVID-19 mortality prediction

After applying different methods of dealing with missing data; the complete case dataset (for the first strategy) and imputed datasets separately were prepared for use in the following steps (Fig. 1).

### Data Description

The quantitative features described as mean ± standard deviation and qualitative features as frequency and percentage. Two independent sample t-test were used to compare the mean of the quantitative features between two groups. To investigate the relationship between the qualitative features in pairs, the Pearson Chi-square test was employed. The significance level was set at 0.05 in all analyses.

### Feature selection using random forest

Due to the large number of input features, the RF algorithm was used as one of the most common approaches to identify important features that had acceptable results. RF is one of the supervised learning algorithms for classification and regression. The RF is an ensemble of several decision trees that grow using recursive partitioning of bootstrap samples. RF uses several indices to calculate the importance of features in predicting outcome, and one of them is the Gini index and is the value between zero to one [24]. In this study, the RF algorithm with 600 decision trees and the Gini index employed to calculate the importance of each feature, and the features with a relative importance value higher than 4% chose for further analysis. Also, the RF was considered as a classification model for comparing with the other models.

### Logistic regression model

Logistic regression is a traditional statistical model used in the classification task. For a binary outcome, the logistic regression model is shown below:

1 $$\mathrm{log}\left(\frac{p}{1-p}\right)={\beta }_{0}+\sum_{j=1}^{q}{\beta }_{j}{x}_{ij}+{\varepsilon }_{ij},$$

where for a given input feature ${x}_{i}=\left({x}_{1i}, \dots , {x}_{iq}\right), i=1,\dots ,n;$ *n* is the number of training samples, *q* is the number of the input features, and p is the probability of belonging to class 1, the logarithm of the odds of this class ($\mathrm{log}\left(\frac{p}{1-p}\right)$) is called the logit function which is a linear function of the input features. Also, ${\beta }_{\mathrm{j}}$ are regression coefficients that are estimated based on the dataset from the maximum likelihood method [25].

### Gradient Boosting Decision Tree (GBDT)

GBDT is a reinforcement algorithm in machine learning where several weak classifiers (individual decision trees) are constructed to form a strong classifier. By combining the results of each weak classifier, the end prediction results are obtained. For a binary outcome, GBDT used the decision tree as the weak classifier and makes global convergence of the algorithm by following the negative gradient [26]. Let ${x}_{i}=\left({x}_{1i}, \dots , {x}_{ip}\right), i=1,\dots ,n$, *n* and *p* are the number of training samples and the number of the input features, respectively, and ${y}_{i}\in {\left\{\mathrm{0,1}\right\}}_{i=1}^{n}$ denoted input feature or binary target. The steps of GBDT are as follows:

Step I: the model β is the initial constant value, for the regression model (${\varvec{Y}}=\beta {\varvec{X}}$):

2 $$F\left(x\right)=\mathrm{arg}\underset{\beta }{\mathrm{min}}\sum_{i=1}^{n}L\left({y}_{i},\beta \right),$$

Step II: calculate the residuals; let ${F}_{m}:{\mathbf{R}}^{p}\to \mathbf{R}$ be a predictive model at iteration m,$m=1,\dots ,M$, and let $L\left({y}_{i},{F}_{m}({x}_{i})\right)$ be a differentiable loss function. According to the least square approach, the parameter ${e}_{m}$ of the model is obtained and the model $h({x}_{i};{e}_{m})$ is fitted.

3 $${e}_{m}=\mathrm{arg}\underset{e,\beta }{\mathrm{min}}\sum_{i=1}^{n}{\left(-\frac{\partial L\left({y}_{i},{F}_{m-1}({x}_{i})\right)}{\partial {F}_{m-1}({x}_{i})}-\beta h\left({x}_{i};e\right)\right)}^{2},$$

Step III: minimization of loss function; where ${\beta }_{m}$ is obtained by fitting a regression tree to the gradients of each sample concerning the current estimator at stage m;

4 $${\beta }_{m}=\mathrm{arg}\underset{e,\beta }{\mathrm{min}}\sum_{i=1}^{n}L\left({y}_{i},{F}_{m-1}\left({x}_{i}\right)+\beta h\left({x}_{i};e\right)\right),$$

Step IV: update of the model and reduce overfitting ($h\left({x}_{i};e\right)$ so called learning rate);

5 $${F}_{m}\left({x}_{i}\right)={F}_{m-1}\left({x}_{i}\right)+{\beta }_{m} h\left({x}_{i};e\right),$$

These steps are repeated until *m* trees are grown [27].

Be noted that, if the target feature was binary, the logistic regression was selected for the growing tree. Therefore, the model β is the initial constant value, for the logistic regression model;

6 $$F\left(x\right)=\mathrm{arg}\underset{\beta }{\mathrm{min}}\sum_{i=1}^{n}-{y}_{i}\mathrm{log}\left({\widehat{p}}_{i}\right)-\left(1-{y}_{i}\right)\mathrm{log}\left(1-{\widehat{p}}_{i}\right),$$

### Generalized additive models

GAMs are a semi-parametric extension of the generalized linear models, used for the case when there is no a priori reason for choosing a particular response function (such as linear, quadratic, etc.). GAMs are interpretable by design because of their functional forms. Given an input ${x}_{j}\in {\mathbb{R}}^{D} j=1,\dots ,p$, where p is the number of features a binary response ${y}_{i} i=1,\dots ,n$, where n is sample belongs to an exponential or non-exponential family distribution, a link function $g$ (e.g. $g$ is $\mathrm{log}\frac{\pi }{1-\pi }$ in binary classification, $\pi$ is the probability of death), main effects ${f}_{j}$ for the *j*^th^ feature called smooth functions with $\mathrm{E}\left({f}_{j}\right)=0$. For a univariate response variable of multiple features, GAMs is expressed as follows [18]:

7 $$g\left(\mu \right)={\beta }_{0}+\sum_{j=1}^{p}{f}_{j}\left({x}_{ji}\right)+\varepsilon ,$$

where $\mu =E\left({y}_{i}\right)$, ${\beta }_{0}$ is intercept parameter, and $\varepsilon \sim N\left(0,{\sigma }^{2}\right)$ is random variables. In GAMs the linear or nonlinear relationships between response and features follow smooth patterns and are explained by unspecified smooth functions known as splines or basis function. The smoothness of each function determined by the smoothing regularization parameter known $\lambda$. In this study, the GAM was fitted by 30 to 300 basis functions, and the $\lambda$ varies from 0.1 to 0.9.

### Deep Neural Networks (DNN)

**Fig. 2:**
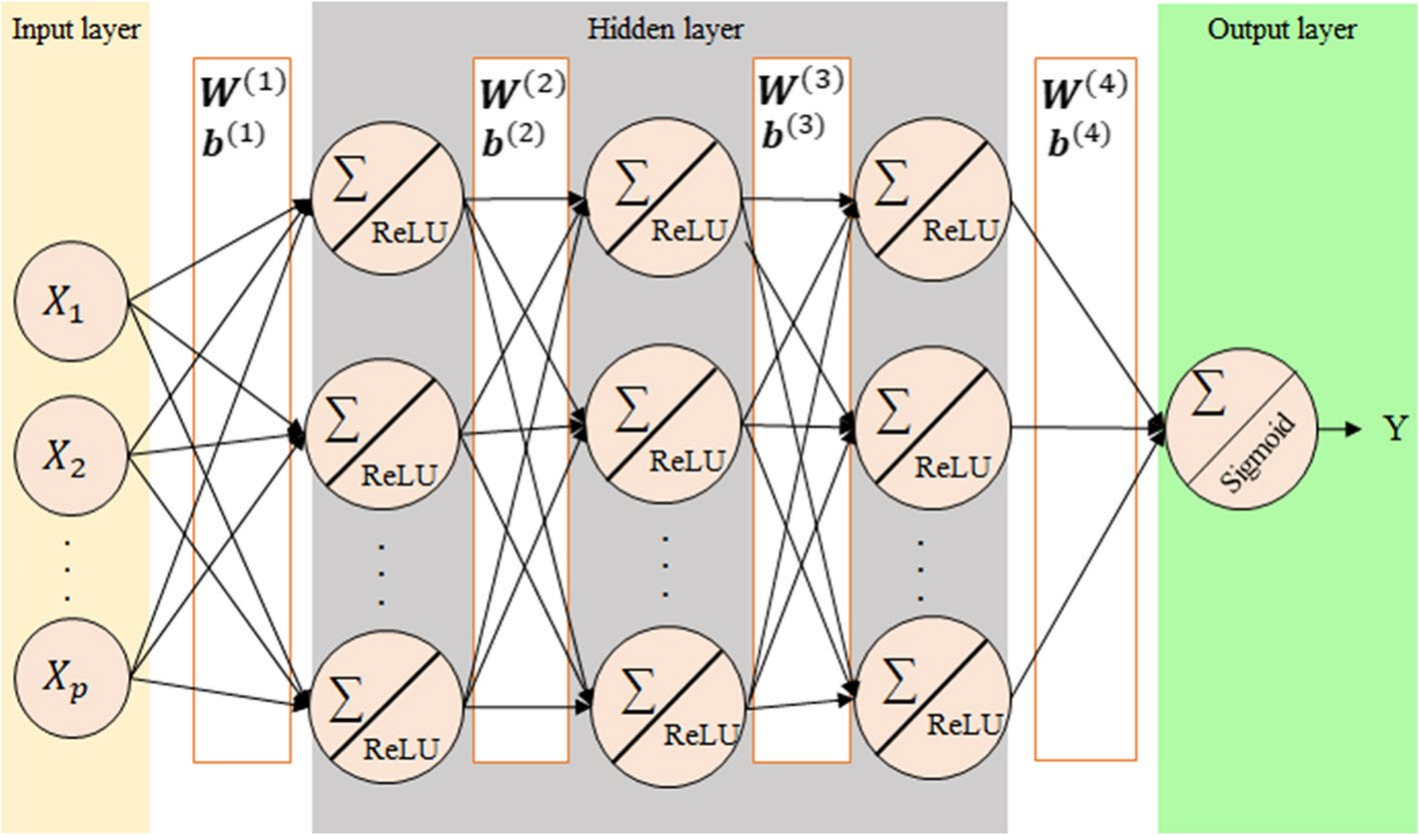
The structure of the DNN

The structure of a shallow neural network consists of three layers, i.e., the input, the hidden, and the output was considered. Each layer has a weight that indicates the effect of the features on each other. The goal of neural network is to reduce the error or cost function in classification or regression and bring the network closer to the desired result. To achieve this goal the connection weights is update during training by various algorithms i.e. backpropagation, and amount of changes in weights control by a hyperparameter called learning rate. Another parameter known as batch size should be obtained which is the number of training examples in one forward or backward pass. In DNNs, the hidden layers are more than one, which may increase the classification and prediction accuracy of the network [28]. Schematic representation of the DNN architecture used in the current study with 3 hidden layers containing 100 nodes in each layer is given in Fig. 2. It should be noted that rectified linear unit (ReLU) activation function used for all hidden layers.

The mathematical form of this structure is given following;

8 $$\mathrm{Y}=\mathrm{sigmoid}\left\{{{\varvec{W}}}^{\left(4\right)}\mathrm{ReLU}\left({{\varvec{W}}}^{\left(3\right)}\mathrm{ReLU}\left({{\varvec{W}}}^{\left(2\right)}\mathrm{ReLU}\left({{{\varvec{W}}}^{\left(1\right)}{{\varvec{X}}}_{{\varvec{i}}}+{\varvec{b}}}^{\left(1\right)}\right)+{{\varvec{b}}}^{\left(2\right)}\right)+{{\varvec{b}}}^{\left(3\right)}\right)+{{\varvec{b}}}^{<span class='reftype'><span class='reftype'>(4)</span></span>}\right\},$$

where **W** matrix and **b** vectors are weight and bias in each layer.

Initial values of **W** matrix was chosen as random values from a normal distribution with mean and variance equal to zero and 0.2, respectively, and the initial values of **b** vector was chosen as **1**.

### Generalized Neural Additive Models

GNAMs belong to the GAMs family and learn a linear combination of multi-layer perceptron (MLP) with an input, an output, and several hidden layers. The output of each MLP is

9 $${f}_{j}\left({x}_{j}\right)={\omega }_{1j}\mathrm{ExU}\left({x}_{j,1}\right)+\dots +{\omega }_{\tau j}\mathrm{ExU}\left({x}_{j,\tau }\right),$$

where ${\varvec{\omega}}$ is the network parameter, $j=1,\dots ,p$, where p is the number of features, and initial values of these parameters could be chosen as random values from a normal distribution with mean and variance equal to zero and 0.2, respectively. It should be noted that the weight distribution was selected based on the pre-trained source network proposed by Agarwal et al., [20] which was designed for a similar binary classification task.

**Fig. 3:**
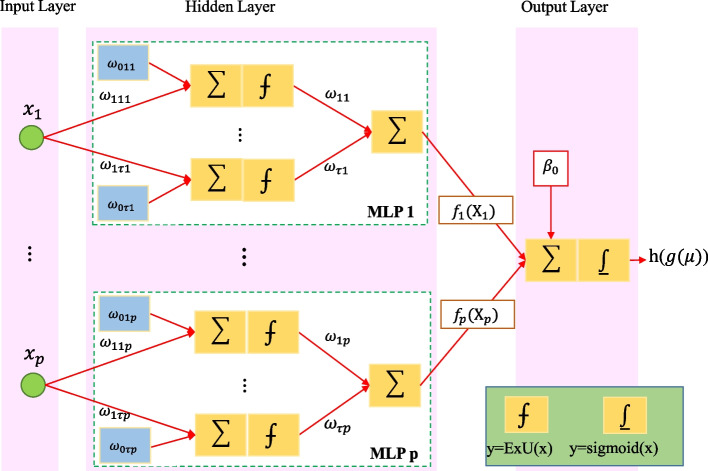
Example of a GNAM architecture

The Exp-Centered (ExU) activation function in the hidden layer for each neuron computes given by

10 $$\mathrm{ExU}\left({x}_{j,\tau }\right)=\left({x}_{j}-{\omega }_{0\tau j}\right)\mathrm{exp}\left({\omega }_{1\tau j}\right),$$

where $\tau =1,\dots ,k$, *k* is the number of neurons in the hidden layer of the *j*^th^ MLP. The sum of all MLP outputs, in addition to the intercept of the model is $g\left(\mu \right)$ which is shown earlier in formula (1). In the last step to classify binary outcome, sigmoid activation function, h(.), was employed [20]. The architecture of the GNAMs with a hidden layer is presented in Fig. 3.

One of the characteristics of the GNAMs is interpreting by visualizing its corresponding smooth function from, ${f}_{j}({x}_{j})$, versus ${x}_{j}$. We take this advantage of the GNAMs and plot each ${f}_{j}({x}_{j})$, versus ${x}_{j}$ for features extracted in feature selection step.

Based on the loss function below, the training of GNAMs is done,

11 $$L\left(\theta \right)={E}_{x,y D}\left(l\left(x,y;\theta \right)+{\lambda }_{1}\eta \left(x;\theta \right)\right)+{\lambda }_{2}\gamma \left(\theta \right),$$

where $D={\{{x}_{(i)},{y}_{(i)}\}}_{i=1}^{n}$ is the training set of size n for input (x) and target (y) features, $l(x, y;\theta )$ is the loss function, $\eta \left(x;\theta \right)=\frac{1}{K}{\sum }_{x}{\sum }_{k}{\left({f}_{k}^{\theta }\left({x}_{k}\right)\right)}^{2}$ is the output penalty for K features (L2 norm), $\gamma \left(\theta \right)$ is the weight decay for K features (L2 norm), ${f}_{k}^{\theta }$ is the feature network for the *k*^*t*ℎ^ feature. The development of the network is also regularized based on feature dropout (drop collinearity features during training) and dropout related to smoothness (regularization of ExUs in each features until the smooth functions are learned while being able to represent jumps) with coefficients λ_3_ and λ_4_ respectively.

Then, the cross-entropy loss function for binary target given as,

12 $$l\left(x,y;\theta \right)=-ylog\left({p}_{\theta }\left(x\right)\right)-\left(1-y\right)\mathrm{log}\left(1-{p}_{\theta }\left(x\right)\right),$$

Where ${p}_{\theta }\left(x\right)=sigmoid\left({\beta }_{0}^{\theta }+\sum_{k=1}^{K}{f}_{k}^{\theta }\left({x}_{k}\right)\right)$ is predicted probability from output GNAMs [20].

Tuning of hyperparameters of Adam optimizer for training networks such as learning rate and batch size were tested based on the values between 0.001 to 0.2 and 100 to 500, respectively. With the aim of avoiding overfitting, the value of regularization parameters (**λ**) for DNN and GNAM was tuned and the optimized values were set as follows: the λ_1_ (output penalty coefficient) in the discrete set {0.001, 0.01, 0.1}, λ_2_ (weight decay coefficient) in the discrete set {0, 0.00001, 0.0001, 0.001, 0.01}, λ_3_ (dropout coefficient) in the discrete set {0, 0.1, 0.3, 0.5, 0.7, 0.9} and λ_4_ (feature dropout coefficient) in the discrete set {0, 0.05, 0.1}. The tuning of the λ_2_ and λ_3_ parameters for DNNs was the same as the tuning of the GNAMs model and the number of epochs for both models was 200. The desired range for mentioned parameters such as the number of hidden layer neurons, learning rate, number of the epoch, etc., was also valued based on the range proposed in Agarwal's study [20]. However, the specific optimal value of each hyperparameter was determined according to the data of the present study by the method of cross-validation.

### Evaluation metrics and Class imbalanced issue

Firstly, regarding the imbalance ratio of 4:10 (minority/majority) for the imbalanced binary classification problem, data balancing was done using the synthetic minority over-sampling technique (SMOTE) method. For subsequent analysis, the dataset is randomly divided into the two subsets of train and test with a ratio of 7:3, respectively. The process of splitting the dataset into the train and test sets was repeated fifty times. The desired prediction models were fitted based on each data set and the evaluation indices for the respective train and test sets were calculated separately. The final performance of the models is calculated as the average of these iterations.

Then the logistic regression, RF, GBDT, GAM, DNN, and GNAM are trained based on the selected features. To compare the predictive performance of models the accuracy, F1-score and the area under the receiver operating characteristics curve (AUC) indices were employed. The steps are shown graphically in Fig. 1. For all evaluation metrics the closer the value to one showed the higher the diagnostic power of the test or the predictive accuracy of the model. The analysis of these methods was done by python 3.8 with sklearn and torch modules using Xeon® 4210 Core i32 CPU with 128 GB ram memory and the source code for NAM models available at https://neural-additive-models.github.io. Although, depending on the conditions and available data, some changes have been made in the original codes.

## Results

In this study, out of 2181 (53.6% male) COVID-19 patients, 1557 were recovered and 624 were dead. The mean age of recovered patients (58.25 ± 16.52 years) was significantly lower than dead patients (71.17 ± 14.44 years) (p < 0.001). Thoroughly, the frequency, percentage, mean, and standard deviation of the mentioned clinical biomarkers with their statistical significance between the two groups of dead and recovered patients are shown in Table 1.

**Fig. 4:**
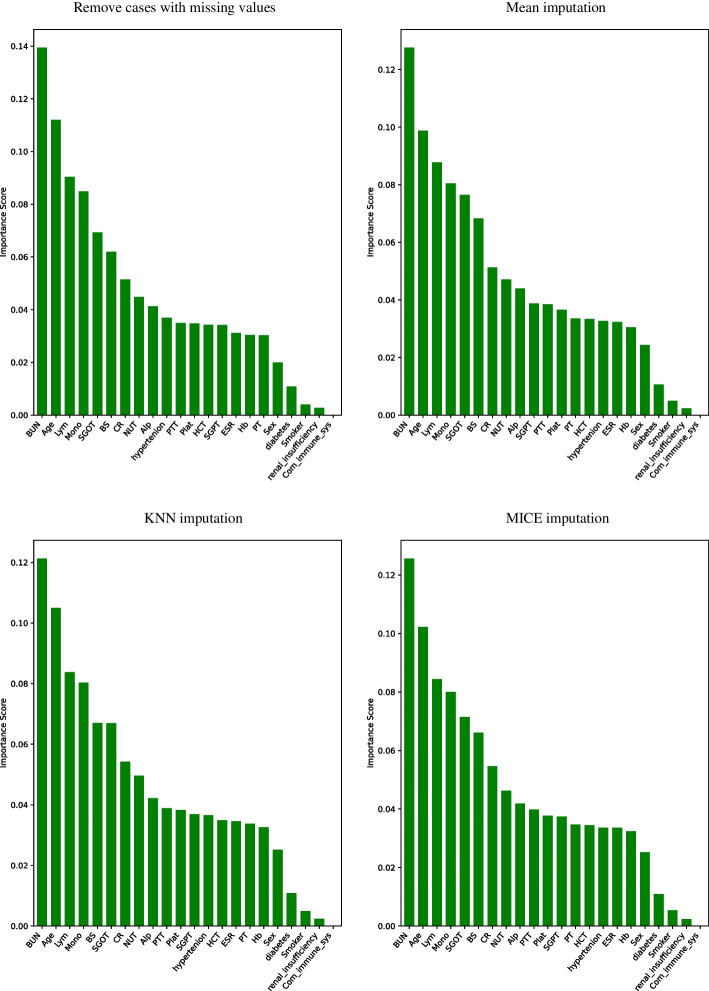
The relative importance of all features selected by random forest classifier based on four imputation strategies (complete case dataset and imputed datasets)

After the implementation of four imputation strategies, the relative importance score of each feature was calculated by Gini index in RF classifier and the results were reported in Fig. 4. These figures confirmed that these different strategies have led to the selection of the same important features; BUN and Com.immune.sys selected as the most and the least important features, respectively. Ten first important features with the relative importance more than 4% were used as the final input features to fit the logistic, GBDT, RF, GAM, DNN, and GNAM models.

The optimal parameters values of different models were adjusted according to cross-validation in different imputation scenarios as follows:

Based on the first strategy of handling missing value (remove cases with missing values); The best results for GAM were obtained by 250 basis functions and $\lambda =0.8$. For the GBDT, the number of trees and learning rate regularization parameters were set to 200 and 0.05, respectively. For the RF, the number of trees and the maximum depth of the tree were set to 300 and 10, respectively.

After various checks with different values of the regularization parameters, the DNN optimized by the λ_2_ and λ_3_ regularization parameters of 0.001 and 0.3, respectively, and the batch size 150, learning rate were set to 0.005, the number of hidden layers in this model containing 3 layers with 86, 64, and 16 neurons in each hidden layer, respectively.

The GNAM was optimized by the batch size of 150, the learning rate were set to 0.005, and the number of hidden layers in this model containing 3 layers with 86, 64, and 16 neurons, respectively. Also, the GNAM optimized by the λ_1_, λ_2_, λ_3_, and λ_4_ regularization parameters were set to 0.01, 0.001, 0.5, and 0.05, respectively.

Based on three imputation strategies similar results were obtained; The best results based on GAM were obtained by 230 basis functions and $\lambda =0.75$. For the GBDT, the number of trees and learning rate regularization parameters were set to 200 and 0.04, respectively. For the RF, the number of trees and the maximum depth of the tree were set to 300 and 10, respectively.

After various checks with different values of the regularization parameters, the DNN optimized by the λ_2_ and λ_3_ regularization parameters were set to 0.001 and 0.25, respectively, and the batch size 300, learning rate were set to 0.002, the number of hidden layers in this model containing 3 layers with 86, 64, and 16 neurons in each hidden layer, respectively.

The GNAM was optimized by the batch size 300, the learning rate were set to 0.002, and the number of hidden layers in this model containing 3 layers with 86, 64, and 16 neurons, respectively. Also, the GNAM optimized by the λ_1_, λ_2_, λ_3_, and λ_4_ regularization parameters were set to 0.01, 0.001, 0.4, and 0.03, respectively.

The accuracy, F1-score and AUC of the trained models based on the four imputation strategies are reported in Table 2. Results showed that, the GNAM model had the best performance with the test accuracy, F1-score and AUC value 0.847, 0.691, and 0.774 for the removed missing values strategy (a), and AUC value of 0.855, 0.704, and 0.782 for KNN imputation dataset, respectively. The GAM model in all datasets had the worst performance with the test accuracy, F1-score and AUC value.

**Table 2:** Accuracy, F1-score and AUC of the different classification models for different imputation strategeis, based on the train and test datasets

IM	Models	Train						Test					
		**Accuracy**		**AUC**		**F1-score**		**Accuracy**		**AUC**		**F1-score**	
		Mean	SE	Mean	SE	Mean	SE	Mean	SE	Mean	SE	Mean	SE
**a**	GNAM	0.885	0.003	0.851	0.001	0.784	0.003	**0.847**	0.002	**0.774**	0.002	**0.691**	0.003
	logistic	0.862	0.004	0.842	0.003	0.743	0.005	0.837	0.002	0.761	0.003	0.681	0.003
	GBDT	0.861	0.003	0.854	0.001	0.783	0.003	0.839	0.002	0.768	0.002	0.684	0.002
	DNN	0.821	0.006	0.808	0.005	0.723	0.004	0.813	0.004	0.742	0.006	0.641	0.003
	GAM	0.721	0.002	0.719	0.002	0.699	0.002	0.782	0.004	0.672	0.003	0.622	0.003
	RF	0.882	0.002	0.862	0.001	0.788	0.002	0.841	0.002	0.771	0.001	0.685	0.002
**b**	GNAM	0.887	0.003	0.860	0.002	0.791	0.003	**0.851**	0.002	**0.779**	0.002	**0.696**	0.003
	logistic	0.869	0.003	0.846	0.004	0.749	0.005	0.840	0.002	0.767	0.003	0.687	0.003
	GBDT	0.884	0.003	0.857	0.001	0.788	0.003	0.845	0.002	0.775	0.002	0.690	0.003
	DNN	0.829	0.006	0.812	0.005	0.728	0.005	0.819	0.005	0.744	0.010	0.645	0.005
	GAM	0.757	0.003	0.721	0.003	0.702	0.002	0.788	0.004	0.679	0.003	0.625	0.003
	RF	0.886	0.002	0.858	0.002	0.793	0.001	0.849	0.001	0.777	0.002	0.691	0.003
**c**	GNAM	0.891	0.003	0.866	0.002	0.796	0.003	**0.855**	0.002	**0.782**	0.002	**0.704**	0.002
	logistic	0.875	0.003	0.854	0.004	0.754	0.004	0.844	0.003	0.772	0.002	0.697	0.004
	GBDT	0.889	0.003	0.864	0.002	0.794	0.003	0.851	0.003	0.781	0.002	0.699	0.003
	DNN	0.834	0.005	0.815	0.006	0.733	0.004	0.823	0.005	0.746	0.009	0.651	0.004
	GAM	0.763	0.003	0.726	0.003	0.705	0.003	0.790	0.004	0.682	0.004	0.629	0.004
	RF	0.890	0.002	0.865	0.002	0.795	0.002	0.853	0.002	0.782	0.002	0.703	0.002
**d**	GNAM	0.890	0.002	0.866	0.002	0.796	0.003	**0.854**	0.002	**0.780**	0.002	**0.702**	0.002
	logistic	0.874	0.003	0.852	0.004	0.753	0.003	0.842	0.003	0.770	0.002	0.694	0.004
	GBDT	0.887	0.003	0.860	0.002	0.794	0.002	0.849	0.003	0.780	0.002	0.698	0.003
	DNN	0.833	0.005	0.811	0.006	0.731	0.005	0.820	0.006	0.745	0.010	0.649	0.004
	GAM	0.762	0.004	0.724	0.003	0.702	0.003	0.789	0.004	0.679	0.004	0.625	0.004
	RF	0.888	0.002	0.864	0.002	0.795	0.003	0.853	0.002	0.779	0.002	0.701	0.002

**a** removed missing values **b** Mean imputed method, **c** KNN imputed method, **d** MICE imputed method

*SE* Standard Error, *GNAM* Generalized Neural Additive Model, *DNN* Deep Neural Network, *GAM* Generalized Additive Model, *RF* Random Forest, *GBDT* Gradient Boosting Decision Tree, *IM* imputation method

**Fig. 5:**
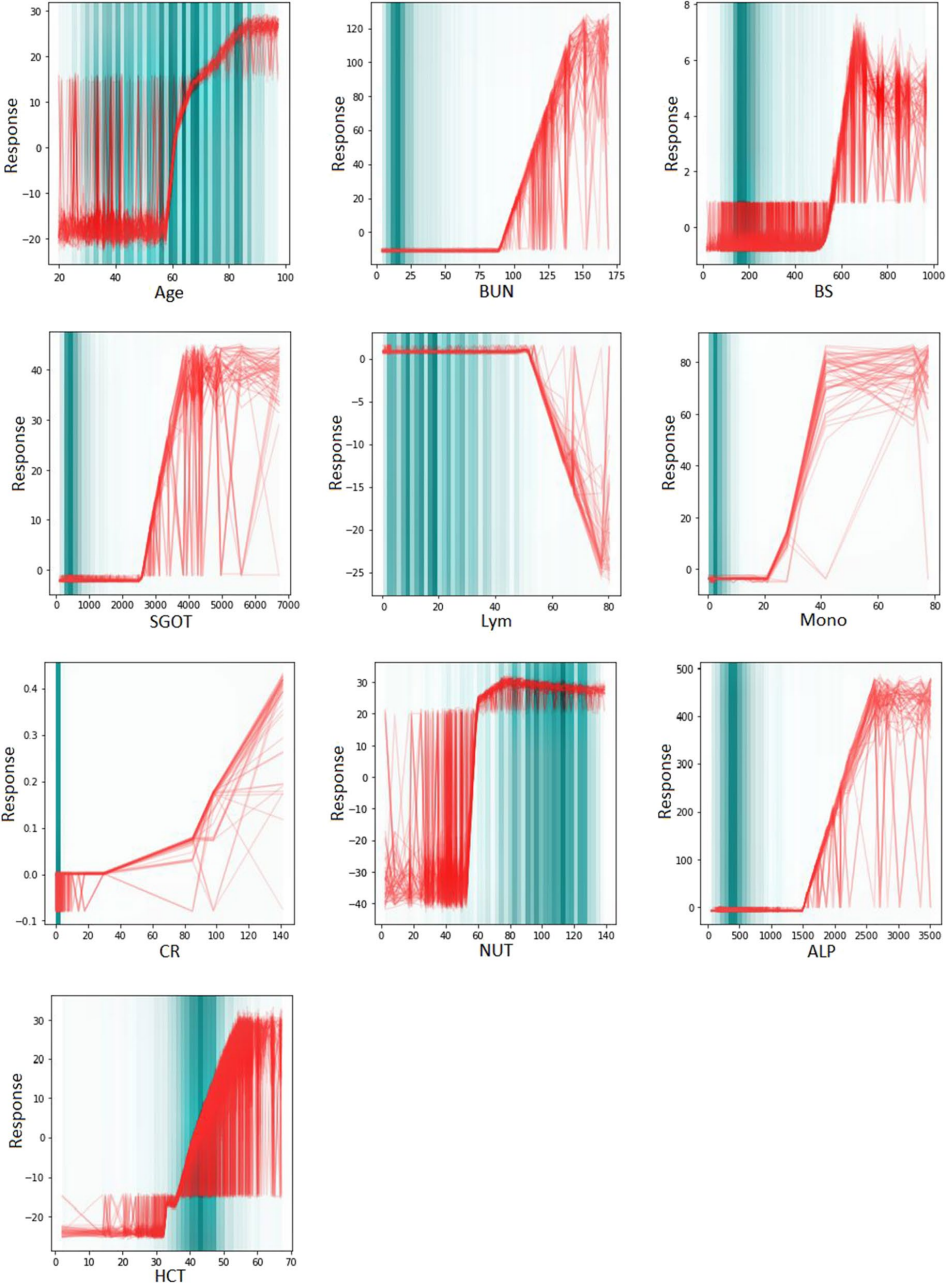
Feature smooth functions learned by ensemble of fifty GNAM along density of COVID-19 dataset (the complete case dataset was used)

Feature smooth functions learned by ensemble of fifty GNAMs along with the density of the COVID-19 dataset be shown in Fig. 5. The deepteal color indicates the data density for each feature. The darker the bar the more data is present in that area and the trend of the learned feature smooth functions is shown by the red lines. For example, the smooth feature function of age showed that the risk of death in COVID-19 patients increased as age increased and for ages, almost over 60 years, the risk of death slope has the most rapid rise.

The smooth feature function of CR showed three different behavior. The risk of death in COVID-19 patients is constant from the CR values zero to 30, it increased from the values 30 to 80, slowly, and for CR almost over 80, the risk of death slope has the most rapid rise.

The smooth feature function of Lym showed, the risk of death in COVID-19 patients is higher in lower values of Lym counts. So by increasing the Lym counts, the probability of death is decreased. It is also showed, the risk of death is high and constant from the values zero to 50, and the risk of death slope has the most rapid fall.

## Discussion

In this study, some effective laboratory findings and demographic features were evaluated to predict the probability of death due to COVID-19 disease. Since the pattern of some features affecting COVID-19 is generally nonlinear, the aim is to determine an appropriate model with the highest prediction accuracy.

The results showed that the risk of death from COVID-19 increases with age. This finding was also confirmed in studies performed on SARS [29], Middle East respiratory syndrome (MERS) [30], and COVID-19 [31]. Because various medical conditions such as hypertension, surgery, hyperlipidemia, and hyperglycemia occur due to old age, this group of patients is susceptible to COVID-19 [32].

Further findings indicated that sex, smoking, compromised immune system, renal insufficiency, PTT, and Plat had no significant effect on death. In Abohamr et al., study, sex, smoking, renal insufficiency, and Plat had a significant effect on death. In the study of Liu et al., smoking, Plat, and PTT factors had no significant effect on death [33]. In the present study, from overall patients, only five patients with a compromised immune system were reported and only one patient experienced death. However, in the study of Kostoff et al., and Yazdanpanah et al., they examined the importance of the immune system feature, and the results of their studies showed that increasing biomarkers of the immune system leads to inflammation and ultimately more damage to other organs and even death [34, 35].

Also, the results of the evaluated factors such as diabetes, hypertension, age, ESR, BUN, BS, CR, PT, SGPT, SGOT, ALP, HCT, Hb, LYM, Mono, and NUT indicate the significant effects on the COVID-19 death that the same finding was reported in the studies of Bertimas et al., Liu et al., Bahl et al., Guan et al., Cao et al., and Chen et al. So that the probability of death increased impressively when increasing their level of the normal range [36–38]. As vital components of the immune system, Neutrophils and lymphocytes play a considerable role in host defense and clearance of infections. In the blood, fewer lymphocyte counts may be an important factor in disease severity and mortality in COVID-19 [39]. Ruan et al. and Chen et al. showed that if factors such as the number of lymphocytes, neutrophils, monocytes, and platelets were out of the normal range, it would indicate virus replication and inflammation in COVID-19 patients [40, 41]. According to studies on the factors affecting the severity of COVID-19 disease, the number of neutrophils in patients with high disease severity was higher than patients with moderate disease severity [42]. In this study, the mortality risk of COVID-19 patients increased with increasing neutrophil count and with decreasing lymphocyte count.

There are several methods to select important features. Based on this method, ten features BUN, Lym, age, BS, SGOT, Mono, CR, NUT, Alp, HCT, respectively, were the most important effective features in the probability of COVID-19 death were selected whose their relative importance was more than 4%. In the study of Ma et al., Lym, age, NUT, Mono [43], in Aljame et al., Lym, NUT, age [44], and in Subudhi et al., Lym, NUT, BS, and age [45] due to the most important risk factors in COVID-19 death was introduced. Also, the results showed that the relative importance of the selected features by removing missing values or imputation did not change much in terms of the order of relative importance.

Many studies, such as the present study, have predicted mortality from COVID-19 using supervised machine learning techniques. The results of predictive performance comparisons showed overlay GNAM had the best performance with the test accuracy with mean accuracy, F1-score and mean AUC in the test dataset of 0.847, 0.691, and 0.774 respectively.

Li et al. considered six important biomarkers (D-dimer, blood oxygen, Lym to NUT ratio, C-reactive protein (CRP), and lactate dehydrogenase) using the DNN model to predict the mortality of COVID-19 patients. Their model AUC was 0.95 [46]. In a study by Lin et al. of 30 demographic and laboratory biomarkers using machine learning methods including neural network to predict mortality of COVID-19 patients, the accuracy and AUC of this model were 0.91 and 0.88, respectively [47]. In the study of Morales et al., ten important demographic and laboratory biomarkers were used, including age, blood pressure, liver, and kidney failure. The accuracy of predicting the death of COVID-19 patients using the neural network model was 0.88 [48].

An important advantage of the GNAM over other neural networks, including DNN (black box), is its interpretability, which is based on the smooth function graphs of each feature learned from the GNAM. In this regard, instead of inflexible parametric assumptions, the relationship between the output and the input feature is expressed by a smoothing function that can be applied to virtually any form of data.

Considering the 3% of missing values, similar results were obtained in different scenarios of handling missing data. The GNAM model compared to other models had a higher predictive performance. Therefore, the interpretable smooth function graphs related to the GNAM model were drawn and reported for the non-missing data. The smooth functions learned from fifty fitted GNAMs are visualized in Fig. 5 to interpret the behavior of each feature. With increasing the value of the features age, BUN, BS, CR, SGOT, ALP, HCT, Mono, and NUT, the smooth function of the logarithm odds of COVID-19 mortality in these features increased. However, with increasing the value of Lym, the smooth function of the logarithm odds decreases.

Since the critical time for disease progression has been reported 10 to 14 days from the onset of clinical symptoms, identifying the factors affecting patient mortality from hospitalization makes it possible for decision-making and predictive power for physicians [41]. Also, the use of interpretable machine learning models such as GNAM, compared to common black-box neural networks, can provide physicians with a broad view of changes resulting from effective variables that reduce or increase the risk of patient mortality. As a result, they increase the accuracy of predicting patient mortality.

## Limitations

The samples used in this study are limited to the central city of Hamadan, Hamadan province, Iran, so the number of generalizable samples was less. To identify the features affecting patient mortality, some patient information such as BMI, predisposing factors, or underlying comorbidities due to high missing did not use. These features may also play an efficient role in predicting patient mortality.

## Conclusion

When the relationships between predictor features and response are nonlinear, machine learning models such as GNAM can perform well in predicting the mortality of COVID-19 patients. Therefore, the use of interpretable machine learning models such as GNAM helps physicians to prioritize some important demographic factors and laboratory findings by identifying the effective features and the type of predictive trend in disease progression.

## Supplementary Information

**Additional file 1.** Source code & dataset.

## Data Availability

The dataset used for analysis during the current study are not publicly available due to restrictions related to our internal review board policy. However, the dataset is available from the corresponding author (Maryam Farhadian) on reasonable request.

## Abbreviations

GNAM: Generalized Neural Additive Model
NAM: Neural Additive Model
RF: Random Forest
MICE: Multivariate Imputation by Chained Equations
GAMs: Generalized Additive Models
SMOT: Synthetic Minority Oversampling Technique
DNNs: Deep Neural Networks
AUC: Area under the Curve
AST: Aspartate Aminotransferase
ALT: Alanine Aminotransferase
LYM: Lymphocytes
NEU: Neutrophil
LDH: Lactate Dehydrogenase
Comp.immune.sys: Compromised immune system
ESR: Erythrocyte Sedimentation Rate
BUN: Blood Urea Nitrogen
BS: Blood Sugar
CR: Blood Creatinine
PT: Prothrombin Time
SGPT: Serum Glutamic-Pyruvic Transaminase
SGOT: Serum Glutamic-Oxaloacetic Transaminase
Alp: Alkaline Phosphatase
PTT: Partial Thromboplastin Time
Plat: Platelets
HCT: Hematocrit
Hb: Hemoglobin
Lym: Lymphocytes
Mono: Monocytes
NUT: Neutrophils
MLP: Multi-Layer Perceptron
ExU: Exp-Centered
ROC: Receiver Operating Characteristics
SE: Standard Error
MERS: Middle East Respiratory Syndrome
CRP: C-Reactive Protein
